# Cooking in the Territorial Medical Service

**Published:** 1911-08-05

**Authors:** 


### The Royal Army Medical Corps Section.

### COOKING IN THE TERRITORIAL MEDICAL SERVICE.

In a previous number of The Hospital we dealt with the subject of field cookery for the men of the Territorial Force, and described the special arrangements that were necessary and the regulation appliances that are furnished for meeting the conditions that pertain to camp life and to service in the field. What was said upon the subject applies equally to the personnel of the field ambulance, who do their own cooking with the camp kettles issued to them, but for the sick and wounded in the ambulance and clearing hospital special cooking is needed. In the present article we propose to outline how this is arranged for, and as it follows the plan adopted in the Regular Army, it may be as well to first briefly outline it. As regards the apparatus furnished to the field ambulances for sick cookery, it consists of a "Primus" stove No. 5 worked with oil, of which three are issued in war time; and a portable stove which consists of two ovens, two boilers with lids, four baking dishes, one grate, and two shelves, capable of cooking for fifty patients. With it all the different cooking methods can be carried out, and as the "Primus" stove will fry, boil, or make beef-tea, it is clear that the field ambulance has suitable equipment for cooking for its sick.

Coming next to the personnel provided, it is laid down that in every tent division of a field ambulance there must be three corporals and three privates with a knowledge of sick cookery, so that there may be with each section of the ambulance one corporal and one private. The training of these men in the R.A.M.C. is not carried out at the Aldershot School of Cookery but in a large Military Hospital, such as the Cambridge Hospital at Aldershot, the Herbert Hospital at Woolwich, and the Queen Alexandra Hospital in London. In each of these hospitals there is a chief or master-cook who is an expert in the matter of sick and invalid cookery and has passed through a course of instruction at the National School of Cookery or has done duty for a time at one of the large London hotels with the view of bringing before him the more refined and attractive side of serving up the food and delicacies of the sick. It is under one of these hospital master-cooks that the R.A.M.C. probationer can obtain his training, and the lines of instruction are laid down in the R.A.M.C. "Training Manual." They provide for a knowledge of the various cuts or joints into which the carcases of beef, veal, mutton, and pork are divided, with hints for guidance in the matter of detecting good from bad quality meat. In the training, too, all the different cooking methods are fully taught, both for meat and vegetables, and practical tuition is given in carving meat as economically as possible. The sterilising of milk and the preparation of a very comprehensive list of suitable dishes and drinks for the sick are also gone through, and this completes a very thorough and practical course of tuition. At the end of it the probationer undergoes an examination, and if he satisfies the examiners he obtains a hospital cooking certificate, which is separate and distinct from the military cooking certificate.

It is upon such lines as these that the six cooks of the tent division of a Territorial field ambulance require to be trained, and the most satisfactory arrangement would be to have them sent to a suitable military hospital and there go through the whole course of instruction. To this, however, the Army authorities would not consent on the ground of expense, as it means that the men would require to get their pay all the time they were attending the hospital; so they meet the case by holding evening courses of instruction, with practical demonstrations in sick cookery, at the School of Instruction.

### Field Cookery in the R.A.M.C.

In connection with this matter of cooking for the sick in a field ambulance, it must be remembered that the latter does not issue diets and that the patients in it are fed from their regiments as long as they are in the ambulance. Were their stay a long one this arrangement would be very inconvenient, but the quick evacuation of the sick from the field ambulance to the clearing hospital is the principle underlying the scheme of medical assistance to the sick and wounded in the field, so that it is in the clearing hospital that a proper knowledge of sick cookery on the part of its personnel will be most necessary. It may be mentioned that, in the case of the field ambulance, should a patient's rations by any chance not be sent over by his regiment the omission must be met by making use of the medical comforts of No. 2 medical or surgical pannier, and an emergency meal in this way provided.

Coming now to the clearing hospitals of the Territorial Force, it will be remembered that these are to be provided and staffed by the voluntary aid detachments that the British Red Cross Society has undertaken to raise and instruct. To carry out the cooking duties it is laid down that of the twenty women who form the rank and file of a woman's detachment four must be qualified as cooks. To ensure their having a proper knowledge of their work they have to attend a course of instruction, and must obtain a certificate from the lecturer to the effect that they have attended regularly and have a competent knowledge of cookery, especially for the sick. From what we have seen of the minimum of instruction required from the members of the voluntary aid detachments we are satisfied that the needs of the sick in this matter are being well provided for and that this department of the work of the clearing hospital is being efficiently organised.

